# Highly conformable terahertz metasurface absorbers via two-photon polymerization on polymeric ultra-thin films

**DOI:** 10.1515/nanoph-2022-0667

**Published:** 2023-02-20

**Authors:** Andrea Ottomaniello, Paolo Vezio, Omar Tricinci, Frank M. Den Hoed, Paul Dean, Alessandro Tredicucci, Virgilio Mattoli

**Affiliations:** Center for Materials Interfaces, Istituto Italiano di Tecnologia, Via R. Piaggio, 34, 56025 Pontedera, PI, Italy; Dipartimento di Fisica E. Fermi, Università di Pisa, Largo Pontecorvo 3, 56127 Pisa, Italy; Engineering and Technology Institute Groningen (ENTEG) , University of Groningen, Nijenborgh 4, Groningen, 4747 AG, The Netherlands; School of Electronic and Electrical Engineering, University of Leeds, Leeds LS29JT, UK; Dipartimento di Fisica E. Fermi and Center for Instrument Sharing of the University of Pisa (CISUP) , Università di Pisa, Largo Pontecorvo 3, 56127 Pisa, Italy

**Keywords:** metasurfaces, nanofabrication, perfect absorbers, terahertz, thin-films, two-photon polymerization

## Abstract

The continuously increasing interest in flexible and integrated photonics requires new strategies for device manufacturing on arbitrary complex surfaces and with smallest possible size, respectively. Terahertz (THz) technology can particularly benefit from this achievement to make compact systems for emission, detection and on-demand manipulation of THz radiation. Here, we present a novel fabrication method to realize conformable terahertz metasurfaces. The flexible and versatile character of polymeric nanomembranes is combined with direct laser writing via two-photon polymerization to develop free-standing ultra-thin quasi-perfect plasmonic absorbers with an unprecedentedly high level of conformability. Moreover, revealing new flexible dielectric materials presenting low absorption and permittivity in the THz range, this work paves the way for the realization of ultra-thin, conformable hybrid or all-dielectric devices to enhance and enlarge the application of THz technologies, and flexible photonics in general.

## 1 Introduction

The full control of light propagation relies on the ability to engineer the interaction with matter at a subwavelength scale. In the general trend to consider systems of asymptotycally decreasing size, metasurfaces, the ultra-thin (quasi-2D) version of metamaterials, offer the most compact and efficient platform to achieve arbitrary manipulation of electromagnetic (EM) waves. Artificial micro- or nano- (metallo or/and dielectric) unit-structures can be arranged in a plane to obtain effective non-standard optical properties for ordinary matter, such as for example magnetism at optical frequencies [1], negative refractive index [2], zero reflection via impedance matching [3], perfect absorption [4], polarization control [5] or wavefront shaping [6]. As applications, a large plethora of metasurface devices have been developed, such as flat lenses, anomalous reflection/refraction deflectors, vortex plates, holograms and invisibility cloaks, just to name a few [7, 8].

Interestingly, their intrinsic two-dimensional character does not only provide compactness for planar integration on complex devices, but it also donates a high degree of structural flexibility and conformability to develop functional artificial “coating” layers. This peculiar property allows to target applications requiring arbitrary complex and curved geometries: for example, to decouple the optical properties of an object from its physical shape [9], to design surface patterns for molecular sensing and cryptography [10, 11] to create conformable holographic displays [12] or more in general to develop free-form devices [13] in the framework of flexible optics and photonics [14]. This demand for flexible, stretchable, non-planar systems and multifunctional materials has been the main lever for the research on innovative fabrication methods in recent years. Conventional micro- and nano-fabrication techniques have thus been oriented towards the exploitation of flexible, thin and/or elastomeric substrates to confer mechanical robustness and versatility to the designed metasurfaces. Depending on the specific operating frequency range, various fabrication techniques have been developed, enabling to build micrometer or nanometer scale elements on non-planar targets [15].

Across the EM spectrum, the terahertz (THz) frequency regime, spanning the range from 0.1 to 10 THz, significantly benefits from mechanically flexible metasurfaces which enhance the potential application of THz technology in fundamental physics studies, imaging, communication and sensing applications [16]. In this regard, a number of flexible devices have already been fabricated, for example to realize wide angle absorbers [17, 18], broadband quarter-wave plates [19] and different sensing applications [20, 21]. All these examples are united by the use of polymer matrices for the metasurface substrate, which offer versatile, large-area and low-cost fabrication. The most used ones are PDMS and polyimide, for their widespread use in flexible electronics, but also parylene, COC, BCB and PMMA and others can be found [22]. Their common characteristics are the low rigidity (low Young’s modulus), low absorption coefficient and low permittivity, which ensure high flexibility, low optical losses and large operating bandwidth, respectively. Onto these polymeric substrates, the resonator patterns are realized with well-established techniques, like conventional photolithography [23, 24] or shadow mask deposition [25], but also other methods such as soft lithography [26], transfer printing [27] and direct laser writing [28] have been demonstrated effective. However, each of these suffers from one or more drawbacks, which mostly limit them to at least several micrometers of thickness, and cause difficulty in transfer feasibility or/and moderate levels of conformability.

In this work, a novel fabrication method for ultra-sub-wavelength free-standing plasmonic metasurface THz absorbers with high degree of conformability is presented. The developed technique merges direct laser writing via two-photon polymerization (2PP) and transfer printing using ultra-thin polymeric membranes. In the 2PP nanolithography technique, femtosecond pulses of a standardly used near-infrared laser are focused inside photosensitive resins able to initiate polymerization at the laser beam spot. Only at this specific volume (called “voxel”) the laser intensity may be high enough to make two-photon absorption events possible triggering the polymer crosslinking, while elsewhere the photoresin is transparent to single photons. By moving the “voxel” inside the material using controlled highly accurate positioning systems, complex full three-dimensional (3D) micro-architectures can be printed in a single step. The threshold non-linear nature of the 2PP process allows beyond diffraction limit resolution of up to 100 nm or better [29]. Thanks to the 3D geometric flexibility and increasing availability of functionalized photoresins, direct laser writing by 2PP has enormous potential for the development of micro- and nano-metamaterials [30]. Here, while the 2PP process is performed to realize the metasurface geometry – the in-plane meta-molecules arrangements and the corresponding thickness – the enhanced spontaneous delamination of a few nanometers thin polyvinyl-formal layer (PVF) via substrate functionalization allows detaching and handling the entire structure after previous metallization. The whole device can be thus straightforwardly suspended or transferred onto a final target using different techniques, which are already demonstrated to allow delamination, suspension and transfer of three-dimensional 2PP structures onto unconventional non-planar surfaces [31]. The optical characterization performed by time-domain spectroscopy reveals that the used polymeric materials, PVF and the specific 2PP photoresin, present low absorption coefficient and permittivity at THz frequencies, thus making them ideal for THz light manipulation. Through a single deposition of a gold layer, two complementary metal patterns displaced by the thickness of the 2PP printed metasurface are created. This two-plane metal resonator distribution allows obtaining high plasmonic absorption resonances. Creating a double metal structure, by coupling a second metallized PVF film via Van der Waals adhesion to the bottom of the same, the optical absorption is strongly enhanced to get quasi-perfect levels in free-standing devices with out-of-plane dimension only a few % of the resonating wavelength. Owing to the ultra-thin thickness and high elasticity of the polymeric materials, objects with radius of curvature as low as tens of micrometers can be easily wrapped with the fabricated metasurfaces. The developed technique thus enlarges the current capabilities of fabricating flexible metamaterials with unprecedented performance in terms of conformability, extending their application in integrated and flexible optics and photonics.

## 2 Fabrication technique

The potential of ultra-thin (thickness less <100nm ) films has emerged over the last decades for the development of fundamental polymer science [32], sensing [33], tissue engineering [34, 35] and biomedical applications [36, 37], and more recently for the development of flexible and wearable electronics [38–42]. Importantly, the fabrication of large area homogeneous freestanding PVF films with extraordinary thin (a few nanometers) thickness has been obtained by functionalizing the substrate to enhance the condition of spontaneous delamination towards nanometric thickness [43]. Exploiting this technique, the possibility to realize conformable freestanding capacitors made of a few tens of nanometers thick PVF membranes were shown [42]. In this case, the polymeric film acts as both structural thickness and dielectric component of the device which is sandwiched inside two-metallic layers to form the ultra-thin capacitors. Interestingly, this double-metal (DM) structure encapsulating a dielectric material has already been extensively used to make EM wave absorbers [44], first for the microwave range and then extending their operation to infrared and visible frequencies.

**Figure 1:**
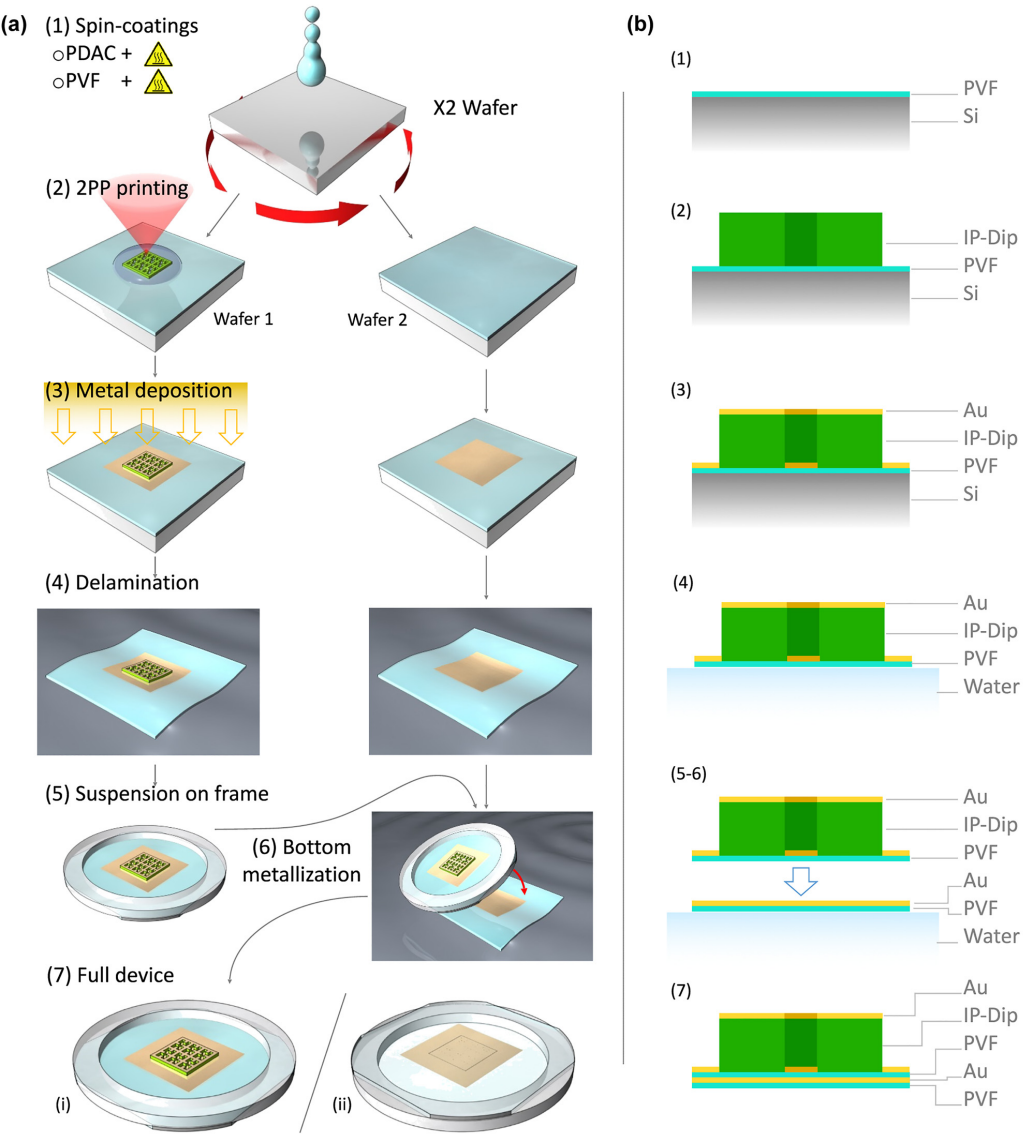
Fabrication process flow. (a) Steps of the fabrication of the ultra-thin conformable metasurface absorbers. (1) Spin-coating of the polymeric thin film on a silicon wafer. Two different wafers are processed in parallel. (2) Printing by 2PP of the IP-Dip cross-linked structure which acts both as dielectric spacer and pattern for the metallization of the device on the first wafer (left sample). (3) Shadow-mask deposition of a 50 nm-thick gold pad on both PVF coated silicon wafers over an area larger than the printed structure. (4) Delamination in water of the polymeric thin film of both wafers carrying the metallized metasurface and the gold pad (left and right samples, respectively). (5) Collection on a holed frame of the delaminated PVF film carrying the metallized 2PP structure. (6) Collection on the same frame of the second PVF film presenting the gold pad which is aligned to that of the metasurface in order to create its bottom metallization. (7) Top (i) and bottom (ii) view of the final device are shown by the left and right sketches, respectively. (b) Schematic profile of the metasurface unit-cell through the entire fabrication process.

Here, we exploit the same DM structure, but including an intermediate dielectric transparent material between the two metallizations which inscribes the metasurface pattern needed for generating the optical absorption resonance in the system. The fabrication procedure is schematized in Figure 1, and it can be summarized in six steps: (1) polymer layer spin-coating, (2) 2PP printing, (3) top metal deposition, (4) delamination, (5) collection and (6) bottom metallization. Two polished silicon wafers are processed in parallel and used as very low roughness substrates to obtain a perfectly homogeneous polymeric thin layer. Exploiting the technique described in [43], a 50 nm-thick PVF layer is created by spin-coating on both wafers after a previous sub-nm functionalization of the surface with a specific cationic electrolyte, which enables the polymeric film to spontaneously delaminate in water. On one of the two coated wafers a 2PP print is performed using a commercial photoresin (IP-Dip, from Nanoscribe) to realize the cross-linked polymeric matrix constituting the designed metasurface structure.

This layer determines both the in-plane geometry of the metasurface and the thickness (from a few 100s nm up to several micrometers) of the dielectric layer of the final absorbers. The device top metallization is then realized by a shadow mask sputtering deposition of a 3 × 3 mm^2^-large 50 nm-thick gold pad which is aligned on top of the 2PP printed metasurface. Covering both the printed and unprinted regions of the metasurface design, two complementary metal patterns displaced by the cross-linked IP-Dip thickness are simultaneously realized by the sputtering deposition (as it will be highlighted in the following section). The same metallization process is also performed for the second PVF spin-coated silicon wafer. The whole device, now comprising the PVF layer plus the metalized 2PP metasurface, is still able to spontaneously delaminate in a water bath, and float on the water tension. The PVF film can thus be easily collected on a specific holed frame, to have the whole device freestanding on the ultra-thin polymeric layer. Finally, also the second PVF layer with the gold pad is delaminated and collected over the same holder. By aligning the metal pad with the metasurface structure, the bottom metallization is obtained and the entire DM metasurface absorbers completed. In the region where the 2PP print is present, the device at the end of the fabrication process is thus structured as follows starting from the bottom layer: PVF thin film, 50 nm-thick gold layer, PVF thin film, IP-dip dielectric with variable thickness and the top 50 nm-thick gold layer. Elsewhere, at the location of the unprinted parts where the development process has completely removed the not cross-linked IP-Dip, the device presents only the PVF plus the gold layers. A sketch of the final sample is shown in Figure 1a at point 7, where the top and back views are shown by the left and right illustration, respectively. In Figure 1b, the material composition of the device throughout the entire process is also reported. All details regarding the fabrication process are reported in the Supplementary Materials.

### 2.1 Device description

**Figure 2:**
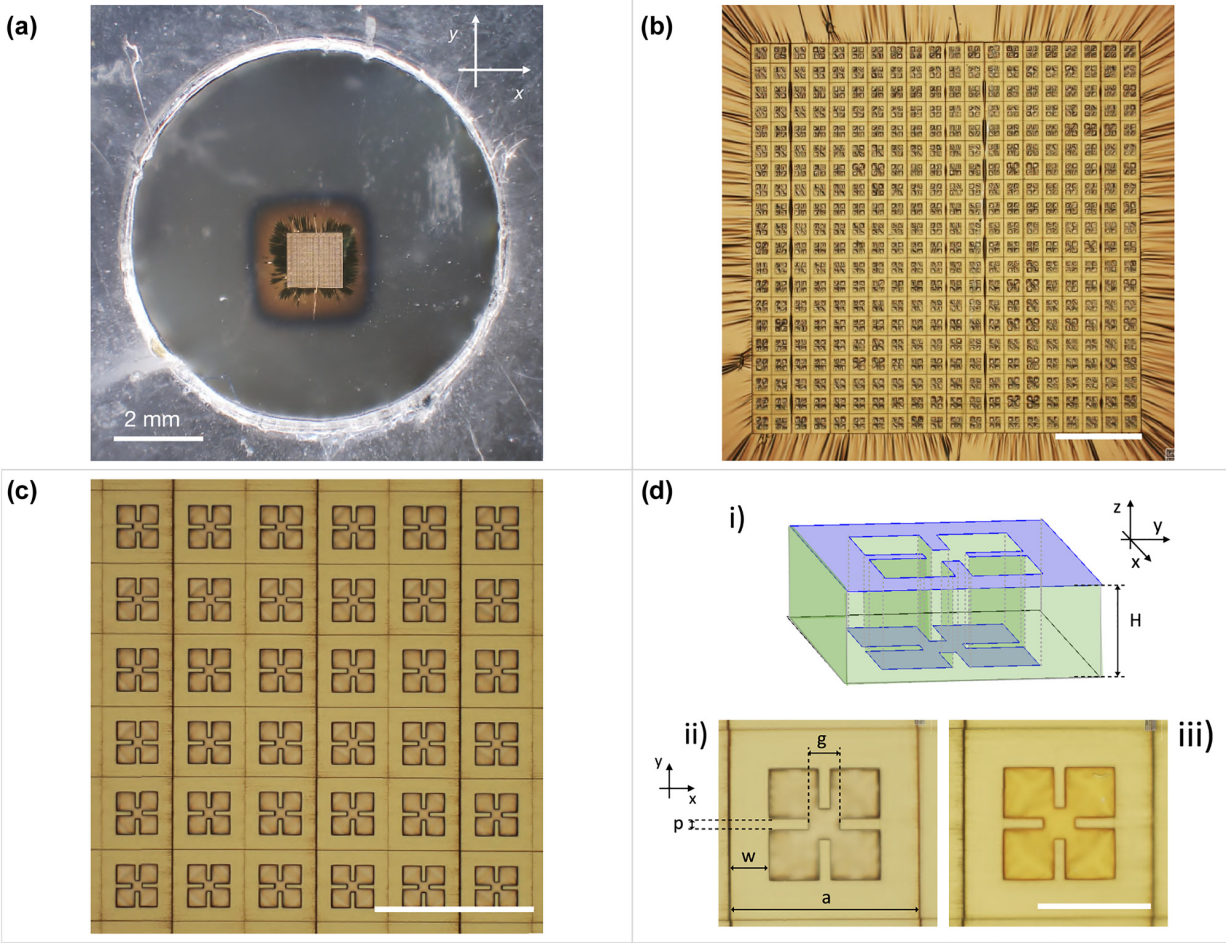
Optical images of a representative fabricated conformable THz metasurface absorber with only the top metallization. (a) Freestanding metasurface absorber on a 50 nm-thick PVF layer after delamination and collection on a holed frame. (b) 2PP patterned region of the ∼2 × 2 mm^2^-large, 2 μm-thick metasurface. (c) Zoom of the metasurface showing the square array of split ring resonators. (d) Scheme of the single metal metasurface unit-cell (i): 3D sketch highligthing (in violet color) the two complementary metal surfaces displaced by the resist thickness (green). The metasurface thickness was scaled by a factor 5 for clarity. Front (ii) and back (iii) view of the metasurface unit-cell of 45 μm-size. The resonator geometrical parameters are reported. Scale bars in (b), (c) and (d) are 200, 100 and 30 μm, respectively.

A fabricated sample presenting only the top metallization is shown in the optical image of Figure 2a. The circular holed frame with the flat suspended transparent PVF membrane can be recognized. At the center, the square 2 μm-thick metasurface absorber is distinguishable from the surrounding unpatterned metallization via a brighter gold color. Figure 2b and c show the metasurface pattern at increasing magnification where it is possible to observe the square array arrangement and geometry of the metasurface unit-cell with size *a* = 43 μm, respectively. The realized resonator shape belongs to the most widely used class of sub-wavelength optical resonators at microwave, THz and FIR frequencies known as split-ring resonators (SRRs), which can present electric and/or magnetic response by design [45]. SRRs-based metasurfaces have been demonstrated to be ideal to achieve also quasi-perfect absorption in DM structures. The working principle of these devices is the engineering of the whole EM response of the system. The dispersions of the effective electric permittivity and magnetic permeability can be independently controlled via the top metasurface design and its coupling with the underneath ground plane (where the induced currents generate the magnetic response), respectively. Interestingly, this can allow the EM impedance of the system to match that of the external medium at a particular resonant frequency or range of operation. This matching indeed ensures that all the power impinging on the metamaterial cannot be transmitted (due to the totally reflective ground plane) nor reflected, and thus it is almost totally absorbed by the system [46]. In this case, the resonator is electric SRRs, presenting LC resonance due to the presence of inductive and capacitive elements constituted by the two perpendicular metal gaps and the corresponding open loops created in the center of the top metallization, respectively. This is shown in Figure 2d (i) where the direct laser printed and unprinted parts of the resonator unit-cell (i.e. the external and internal part, respectively) are highlighted via a 3D sketch. Importantly, as the metal deposition covers both printed and unprinted surfaces, the metal deposition directly creates two aligned and complementary metal patterns in the system: one at the top of the cross-linked photoresin and one in the internal (not-printed) part displaced by the metasurface thickness, which directly lies on the supporting polymeric membrane. This can also be noted in Figure 2d (ii) and (iii), where the top and back view of the unit-cell is shown, respectively. From the top, the internal part of the resonator is observed to be out of focus, while the entire surface of the resonator is in focus from the back. This reveals the good flatness of the PVF freestanding layer carrying the metasurface after the whole fabrication process. The geometrical parameters of the SRR geometry are also reported in the same image. Their values are: *p* = 2 μm, *g* = 5.5 μm, *w* = 7 μm and *a* = 43 μm. Importantly, the metasurface unit-cell presents a *C*_4_-symmetry which provides a polarization independent response at normal incidence.

## 3 Optical characterization

**Figure 3:**
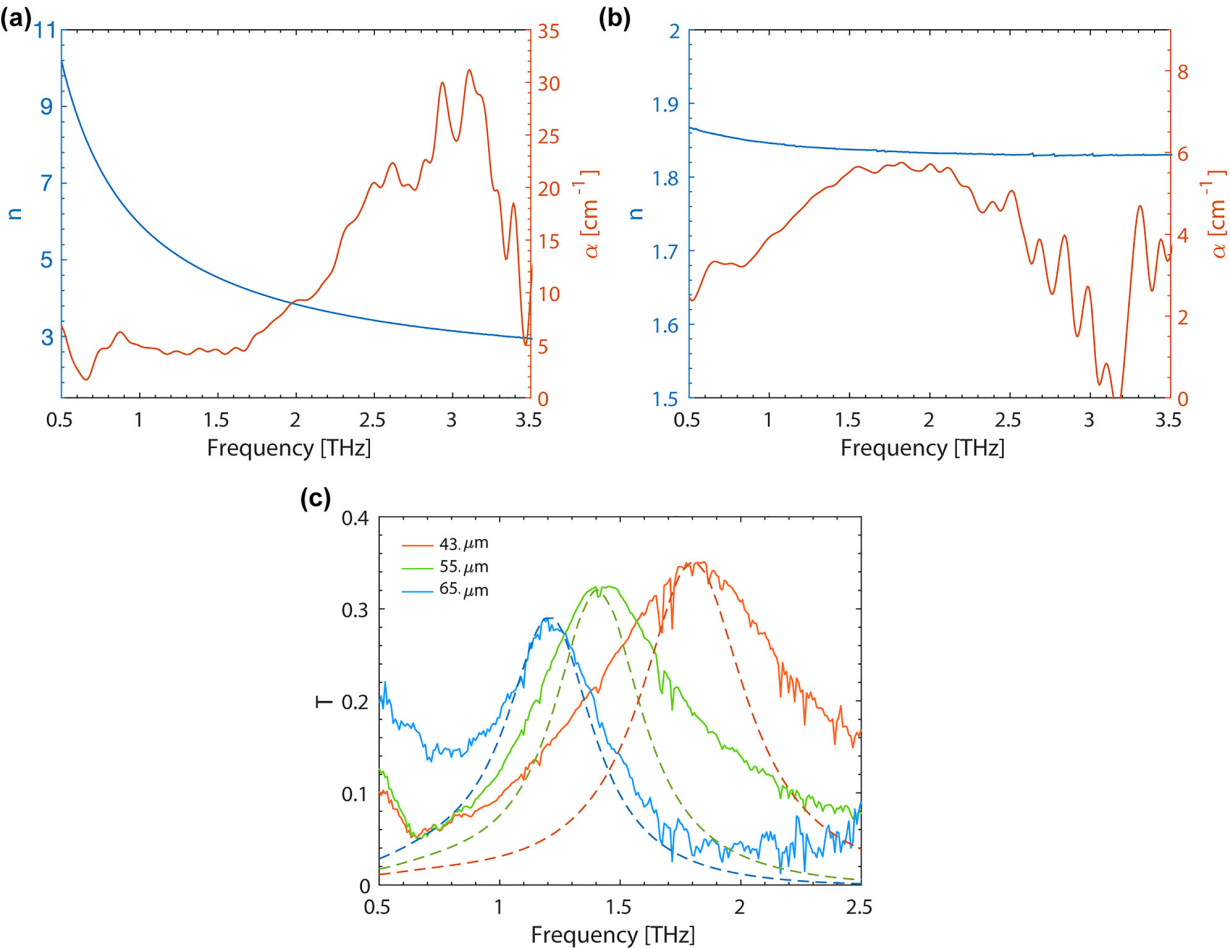
TDS characterization of PVF, IP-Dip and metasurface absorbers. Refractive index (*n*, blue curve) and absorption coeffcient (*α*, red curve) of PVF (a) and IP-Dip (b). (c) Transmission spectra for three metasurfaces having only the top metallization with 43, 55 and 65 μm unit-cell represented by the orange, green and blue solid curves, respectively. Dashed lines with same darker colors report the corresponding calculated results obtained via full-wave finite-element simulations.

In order to realize and design the metasurface absorbers at THz frequencies, the optical properties of the constituent materials were studied. In our case, the attention was focused on the two used polymers which are initially characterized in this frequency region: PVF and IP-Dip. The measurements were performed using time-domain spectroscopy (TDS) in the frequency range from 0.5 to 3.5 THz. The apparatus working in transmission configuration allowed to retrieve the refractive index *n* and absorption coefficient *α* from the amplitude change and phase shift of the transmitted signal with respect to the reference one. In Figure 3a, *n* and *α* dispersion of a suspended PVF thin film are shown. In order to obtain a measurable change with respect to the unperturbed reference signal, a 400 ± 5 nm-thick membrane was employed after spin-coating, delamination and collection on a holed frame. The refractive index is found to vary from 10 to 3 decreasing the frequency, while the absorption is low $\left(<5\;{\text{cm}}^{-1}\right)$ in the range from 0.5 to ∼1.7THz , after which it starts to increase up to 30 cm^−1^ at 3.5 THz to fall again towards low values. The optical properties of the cross-linked IP-Dip were retrieved from a 3.6 μm-thick large 2PP layer which was previously printed on a 50 nm-PVF layer in order to be delaminated and suspended. In this case, the transmitted amplitude from the sample was normalized to that of the PVF membrane used as freestanding support for the 2PP print, and its phase delay calculated by subtraction of the phase accumulated through the same PVF layer. Figure 3b shows the results of the TDS analysis for the printed IP-Dip sample. An almost flat dispersion of the refractive index is observed from 1 to 3.5 THz with a value of ∼1.83. Throughout all the investigated range the absorption coefficient is found to be $<6\;{\text{cm}}^{-1}$ , revealing this material as ideal to be exploited in the THz range as a transparent, low permittivity dielectric.

Metasurfaces with different sizes of the unit-cell were realized exploiting the developed fabrication technique. Samples without the ground metal plane (processed until point 5 in Figure 1) were first characterized using the TDS set-up. The measurements were performed at atmospheric pressure in a dry air purged sample chamber to reduce its humidity concentration. A 2 mm diameter pin-hole is fixed in front of the sample in order to analyse only the transmitted signal from the metasurface region. A reference signal was measured with the same pin-hole aperture before the transmission measurements of each sample. The measured transmittance of three devices with *a* = 43 μm, 55 μm and 65 μm are shown in Figure 3c. The dielectric (IP-Dip) thickness of all samples is *H* = 2.5 μm, while the PVF film and the gold layer are 50 and 40 nm-thick, respectively. A low Q-factor (∼2) resonance is observed for each sample which presents a linear blueshift of frequency peak according to the downscaling of the metasurface unit-cell. The small dispersion of the absorbing materials in the system in fact guarantees an inverse proportionality between the resonance frequency and the size of the meta-atom owing to the linearity of Maxwell’s equations. The oscillations that can be observed in the experimental curves originate from both the set-up noise and possible fluctuations of humidity concentration between the background and sample measurements. Finite-element calculations were performed to simulate the full-wave interaction of linearly polarized EM waves with the metasurfaces. Thanks to the C_4_-symmetry of the metasurface unit-cell, the simulation results allow to obtain the complete EM response at normal incidence considering an EM wave polarized along the *y*-axis. For the PVF layer and the 2PP photoresin we inserted the optical properties measured by the TDS analysis, while for gold a flat dispersion with real and imaginary part equal to 225 and 320 are considered, respectively. Complete details of the performed simulations can be found in the Supplementary Materials. The simulated transmittance for the three samples is reported in Figure 3c as dashed lines for comparison with the experimental data. A very good agreement is found for the resonance peak frequency and amplitude. The slight increase in the transmittance amplitude at resonance for decreasing unit-cell dimensions can be qualitatively explained by a lower EM confinement in the microcavity system. As only the in-plane metasurface geometry is varied while keeping fixed the microcavity thickness, the coupling between the two coupled complementary metasurfaces can vary. From simulations an overlap factor of the time averaged EM energy density with the microcavity is calculated to progressively decrease from a value of 0.28 to 0.25 and 0.22 passing from the 65, to 55 and 43 μm unit-cell metasurfaces. This finally justifies the observed trend in the transmittance amplitude due to a lower absorption in the microcavity. The simulated Q-factors (∼2.8) result higher with respect to the experimental ones. This discrepancy can be ascribed to the possible presence of metal deposition on the internal side-walls of the SRR which can produce an overall increase of the resonance broadening in the system. The deviation from experimental data which can be observed at frequencies lower than 0.7 THz may come from a possible dispersion of the sputtered gold with respect to the constant refractive index we assumed in the simulations.

**Figure 4:**
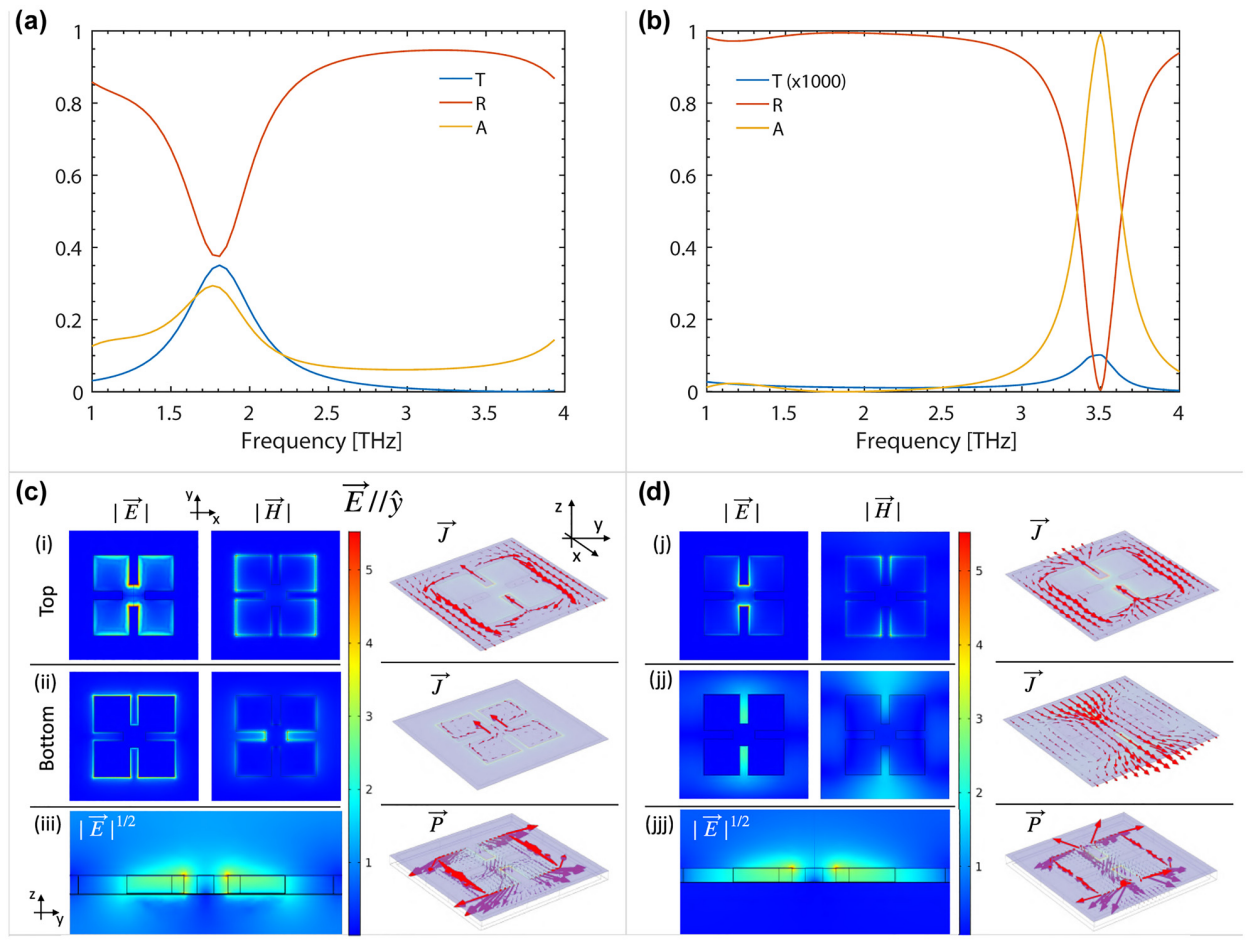
Simulated transmittance, reflectance and absorbance for a metasurface absorber with single (a) and double (b) metallization, with unit-cell size *a* = 43 μm. (c) Electric $\left(\vec{E}\right)$ and magnetic $\left(\vec{H}\right)$ fields distribution, the corresponding current density $\left(\vec{J}\right)$ and Poynting vector $\left(\vec{P}\right)$ in the unit generated by a normally incident EM wave polarized along the *y*-axis $\left(\vec{E}\|\hat{y}\right)$ at the frequency resonance 1.8 THz, in the case of a single metal deposition without ground plane. (i) And (ii) report the $\left|\vec{E}\right|$ , $\left|\vec{H}\right|$ (2D color plots) and $\vec{J}$ (3D arrow plot) for the top and bottom metasurface surfaces. (iii) $\vec{E}$ -distribution in the *zy*-plane (color plot) and corresponding $\vec{P}$ (arrow plot). (j), (jj) and (jjj) Show the same fields with the addition of the ground metal plane underneath the metasurface at the resonance frequency at 3.5 THz. The scale bar indicates the amplitude of |*E*| (V/m) and |*H*| (A/m) with a scale factor of 10^7^, 10^4^ and 10^3^ in the color plot of (i/j), (ii/jj) and (iii/jjj), respectively.

**Figure 5:**
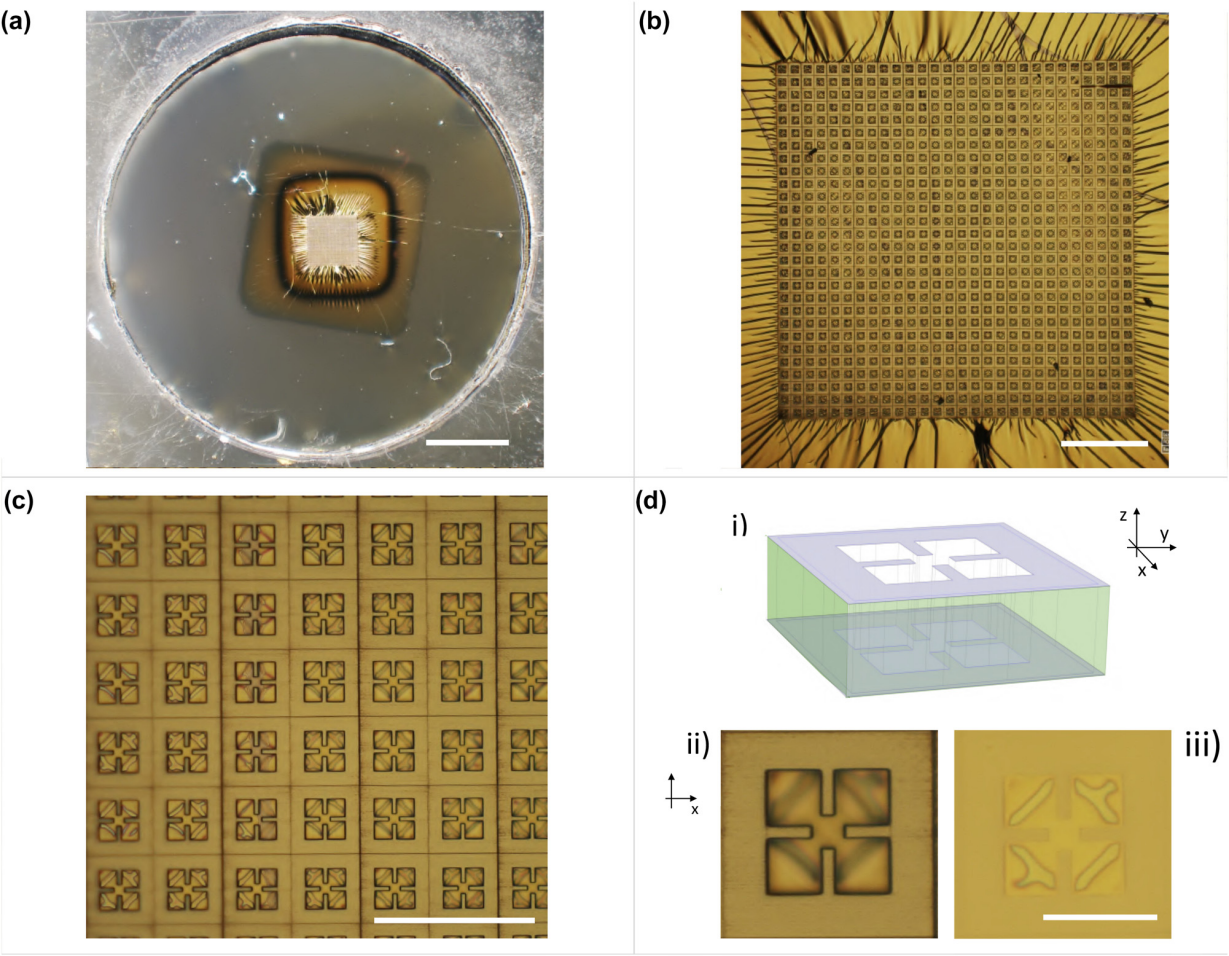
Optical images of a conformable THz metasurface absorber where the top ground plane was added to form the DM-structure. (a) Freestanding DM absorber on the ultra-thin PVF layer at the end of the fabrication process. (b) Image of the 2 × 2 mm^2^ central region of the sample where the metasurface is located. (c) Zoom (700×) of the metasurface. (d) Scheme of the DM metal unit-cell (i): 3D sketch highlighting (in violet color) the top and bottom metal surfaces displaced by the resist thickness (green). The metasurface thickness was scaled by a factor 5 for clarity. Front (i) and (ii) back view of the metasurface unit-cell of 43 μm-size (×3000). Scale bars in (a), (b), (c) and (d) are 2000, 200, 100 and 30 μm, respectively.

The comparison between the EM response of a SM metal metasurface and a DM device, i.e. presenting a 50 nm-thick ground plane metallization provided by the last fabrication step, is then investigated. The performed simulations of a SM and DM metasurface with *a* = 43 μm are shown in Figure 4a and b, respectively. These devices correspond to the samples shown in Figure 2 (SM device) and Figure 5 (DM device). The scattering matrices for light propagation are computed and reported as transmittance, reflectance and absorbance in the system. In agreement with the previously shown TDS measurements in Figure 2c (green curve), the SM device presents an absorption resonance around 1.8 THz with *Q* ∼ 3 and a peak absorbance of 28%. The in-plane EM field distribution (shown in Figure 4c (i)) at resonance can be observed to correspond to the excitation of the LC mode of the SRRs metasurface. The out-of-plane electric field *E* induced current density *J* in the top and bottom metallization patterns and Poynting vector *P* are also reported in Figure 4c (ii) and (iii), respectively. The relatively high absorption level can be explained by the built-in presence of two-complementary metal patterns (see Figure 4d) generating induced currents which are counter-circulating in the top and bottom gold layers displaced by the dielectric with thickness *H* = 2.5 μm. However, even if the coupling between the top and bottom metal patterns is able to produce an additional microcavity effect with high concentration of the EM field in the freestanding device (see Figure 4a (iii)), the incident power is largely reflected (37%) and transmitted (35%) by the system. Interestingly, when a ground metal plane is instead added to the system, the near-field distribution imposed by the coupling of the top metasurface with the uniform bottom metallization allows to achieve the targeted impedance matching between the system and the external medium (air) at a specific metasurface thickness (i.e. dielectric thickness). A quasi-perfect (99.5%) absorption peak with *Q* ∼ 12 emerges at 3.5 THz with a very low amount of reflected power (<0.5%) , and approximately zero transmittance owing to the bottom homogeneous metal mirror. The generated EM fields distribution and the corresponding induced current density at the level of the top metallization (i.e. metasurface pattern) shown in Figure 4d suggest that the observed resonance still arises from the metasurface LC mode. However, the enhanced EM field confinement in this case (as can be clearly observed comparing Figure 4c (iii) and d (jjj)) provides a reduction of the microcavity mode volume from a calculated value of 95 to 49 μm^3^ passing from the single-to the double-metal microcavity, which inevitably leads to the blueshift of the resonance. Importantly, the quasi-perfect absorption due to a joint contribution of both Ohmic and dielectric losses can be obtained in a freestanding device of total thickness which is only a ∼3% of the resonating wavelength.

The fabricated DM absorber with the same metasurface geometry and out-of-plane structure of the simulation to obtain the perfect-absorber at 3.5 THz is shown in Figure 5. In Figure 5a, the whole device suspended on the PVF layer can be seen. The larger square metallic pad having less bright gold color constitutes the underneath metal plane which is separated from the IP-Dip printed layer by two coupled 50 nm-thick PVF film. A very good and uniform adhesion between the first PVF layer carrying the metallized metasurface and the second one providing the bottom metal plane can be observed. This is highlighted also in Figure 5d (i) and (ii) where the top and bottom views of a single unit-cell are reported. Again the perfect Van der Waals adhesion ensures a very flat bottom surface guaranteeing agreement between the experimental and nominal distance of the top metasurface from the bottom ground plane.

**Figure 6:**
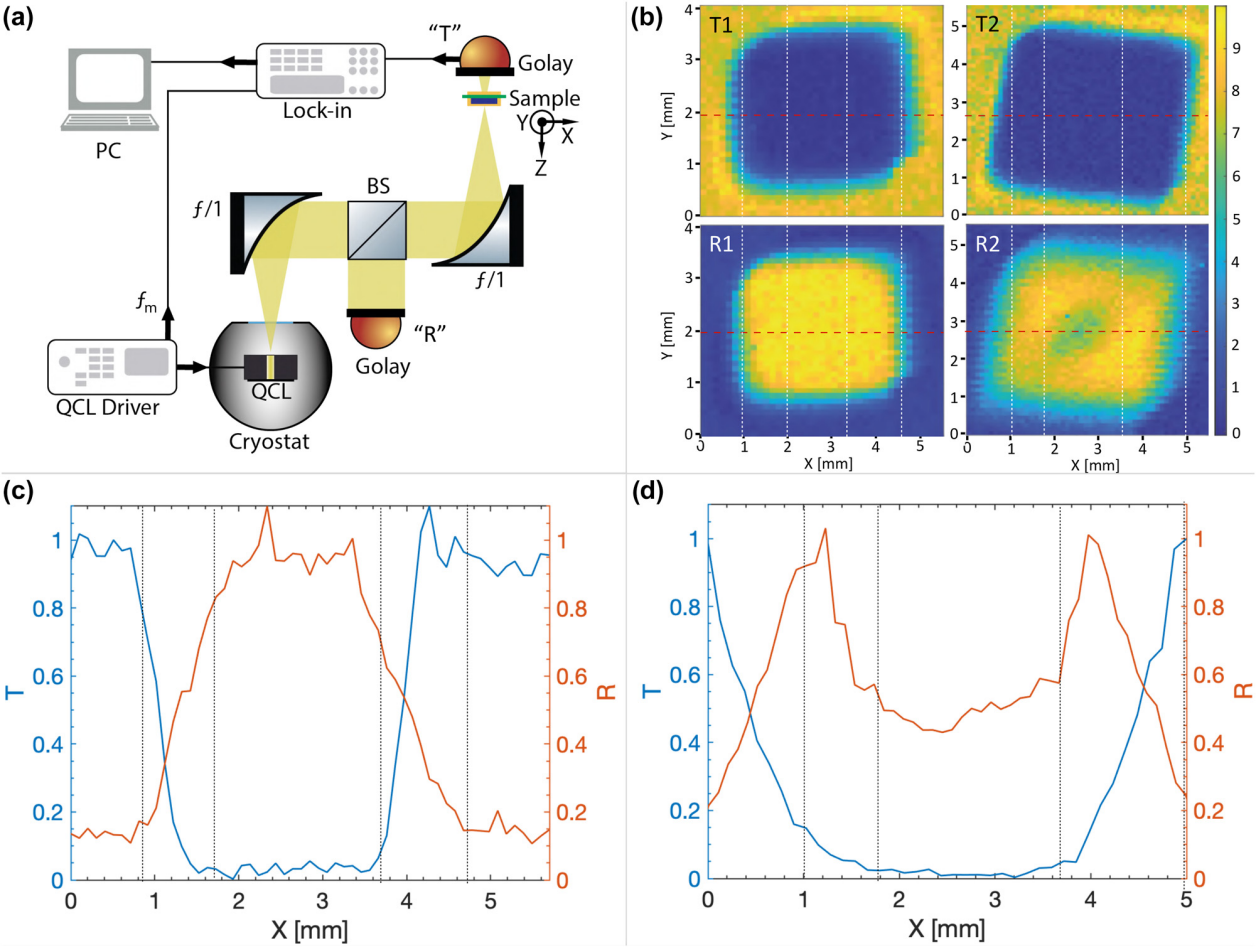
Imaging of the single and DM metasurfaces using a THz quantum cascade laser (QCL). (a) Scheme of the imaging apparatus. The emitted beam of a THz QCL (operating at 78 K and driven in pulse mode at 10 kHz with 2% duty cycle) is collimated and focused on the sample using two off-axis parabolic mirrors with 50 mm focal length. A Golay cell is used to detect the transmitted and reflected power from the sample by fixing it in position “*T*” and “*R*”, respectively. In reflection configuration, a beam splitter is fixed in between the two mirrors. The detected signal is electrically demodulated at *ν*_m_ = 17 Hz using a lock-in amplifier. (b) Transmission (*T*) and reflection (*R*) images of a single metal (1) and a DM (2) metasurfaces with 43 μm unit-cell. (c) And (d) show the transmission and reflection profiles along the *x*-direction (reported in (b) as dashed red lines) for the single- and double-metal metasurfaces, respectively. Vertical dashed lines in (b) and (c) indicate the interface between different materials.

In order to investigate the absorption in the fabricated devices, a single-mode quantum cascade laser (whose active region and operation details are reported in [47]) emitting at the metasurface resonance peak is used to measure the transmitted and reflected power from the freestanding SM and DM devices. The exploited apparatus is shown Figure 6a. A quantum cascade laser is powered in pulse mode at 10 kHz with 2%-duty cycle with an injected current of 1.4 A and kept at a temperature of 78 K by nitrogen liquid cooling to provide stable single-mode emission at ∼3.55THz . Through two parabolic mirrors the THz beam is focused with a 200 × 200 μm^2^-spot onto the sample which is mounted on a three-axes motorized stage in order to control its position with less than micrometer precision. A Golay cell is exploited to detect the transmitted/reflected intensity from the metasurface. A frequency modulation of *ν*_m_ = 17 Hz is imposed to the QCL driving pulse as a reference signal to demodulate via a lock-in amplifier and acquire the detected signal. At focus the sample position is swept in-plane perpendicularly to the direction of the laser beam to perform imaging on the samples. When performing images in transmission the Golay cell is fixed at a location behind the sample, while for reflection imaging a beam-splitter is inserted in between the two mirrors and the Golay cell positioned at a certain distance in the orthogonal direction. Figure 6b shows the acquired transmission and reflection images of the SM and DM samples. They are supposed to provide THz counterparts of the optical images of the SM and DM samples shown in Figures 2a and 5a, respectively. For the SM device, as it is simulated to provide >95% reflected power and almost-null transmission, the whole device is accordingly observed to provide high reflection (null absorption) over the entire sample metallization. No variation of the signal is observed passing from the patterned metasurface of the sample to the surrounding homogeneous region as they behave as nearly perfect THz mirrors. The beam instead starts to be totally transmitted when it impinges on the uncoated PVF layer which is almost completely transparent. The signal profile along a *x*-parallel line crossing the sample along its entire width is reported in Figure 6c, which highlights the interfaces between different transparent/reflective materials. The transition range from the totally transparent to the totally reflective surface is due to the thickness gradient of the gold layer deposited by sputtering with the shadow mask technique (this is also appreciable in the optical images of the samples, Figures 2a and 5a). In the case of the DM sample, the transmission image accordingly shows a uniformly null transmission corresponding to the gold metal coated region of the sample. The reflection image instead presents a significative reduction of the reflected signal at the center of the sample. This can only be ascribed to absorption at the specific wavelength of operation. The good agreement between the geometrical dimension of the metasurface and that emerging from the THz image confirms the identification of the low reflective region in the measurement with the metasurface absorber area. The corresponding profiles of the reflected and transmitted signals shown in Figure 6b highlight the absorbing character of the metasurface placed in the middle of the scan. Even if a considerable (>40%) absorption is observed, the deviation from a quasi-perfect absorption can be ascribed from a possible frequency resonance detuning between the laser emission frequency due to a possible discrepancy between nominal and real geometric parameters of the structure.

**Figure 7:**
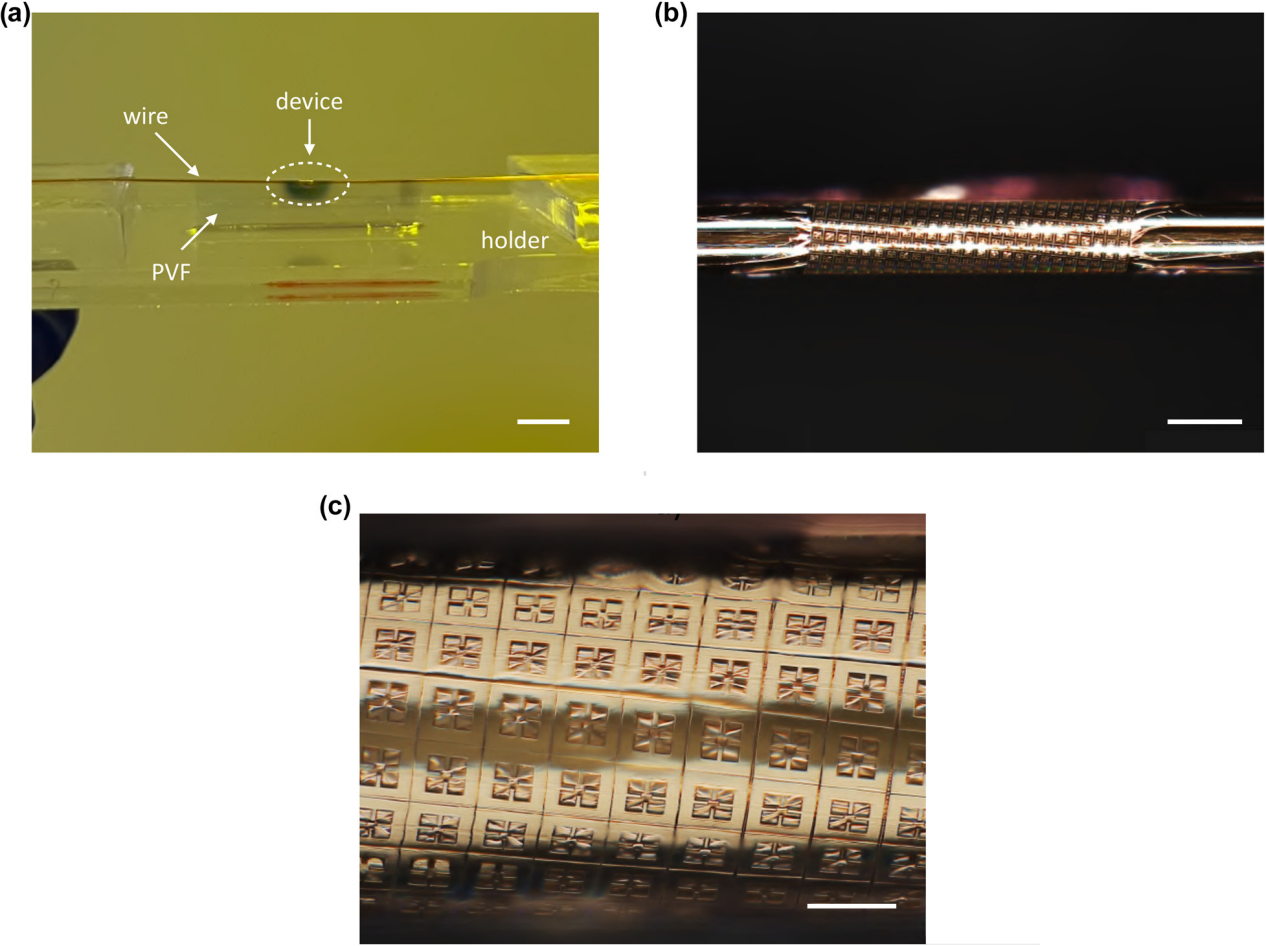
Demonstration of transfer on a high curvature target. (a) Optical image of the holder with the suspended copper wire wrapped by the THz metasurface aborber. (b) Image of the THz absorber showing the all-round conformability on the 100 μm-radius wire surface. (c) Zoom (700×) of the conformable device. Scale bars in (a), (b) and (c) are 5 mm, 250 *μ*m and 50 *μ*m, respectively.

The degree of conformability of the developed metasurfaces was finally tested by transferring them on the cylindrical surface of metal wires. Metasurfaces with dimensions slightly larger than the circumference of the wire targeted for the transfer were thus fabricated. For the collection process on the wires we exploited the water transfer technique developed in [31]. This is performed by using a specific holder (reported in Figure 7a) where the metal wire is suspended in such a way to create a few millimeters high gap between the wire and the bottom surface of the holder. The PVF area enclosing the device to be delaminated is defined to have a side larger than twice this gap by applying a doctor blade to the spin-coated silicon wafer (as shown in the Supplementary Materials). After the delamination process of the PVF film, by immersing in water the holder carrying the suspended wire it is possible to collect the delaminated device floating on the water surface directly on top of the metal wire. During this process the water tension is able to create sufficient strain in the film through capillary forces to conform the metasurface to the wire cylindrical surface. The Van der Waals interaction of the polymer with the surface finally guarantees a very good and stable adhesion. Importantly, the defined gap is fundamental to avoid the PVF film to wrap multiple times on the wire surface during collection by the adhesion of film tails on the holder ground. The result of the water transfer of the film is shown in Figure 7a. At this point, the PVF ultra-thin layer in excess (i.e. not covered by the device metallization) can be easily removed by a standard descum process through oxygen-plasma etching (30 W for 60 s). Figure 7b and c show the perfect conformability of a SM device transferred on a 100 μm-diameter metal wire. The same transfer can be performed also for the DM device by performing two different transfers in series: one for the PVF carrying the bottom metallization and the second one to transfer the SM metasurface. Thanks to the ultra-thin fabricated metasurfaces and exploiting the developed water-based transfer technique, very low radii of curvature can be targeted. In our study, we in fact limited the conformability investigation to a curvature radius which was reasonable for the specific operating wavelength and corresponding resonator size.

Other demonstrations of high curvature conformability are obtained with metasurface absorbers with smallest unit-cell dimension (from 5 to 7 μm, resonating in the atmospheric window 23–37 THz) in order to be easily characterized in reflection by a Fourier-transform spectrometer equipped with an optical microscope to perform spectroscopy in a microscale range (see Supplementary Materials). On metasurfaces resonating around 25 THz, the absorption resonance frequency and amplitude are demonstrated to be not clearly affected by curvature indicating a limited dependence of the metasurface EM response at resonance from their curvature in the case of unpolarized incoming light. Moreover, the all-round transfer on cylindrical surfaces was successfully performed up (but not limited) to a minimum radius of curvature of 12.5 μm. This result demonstrates that, according to the specific operation frequency, the working flexibility of the developed conformable metasurfaces is only limited by the geometric metasurface design with respect to the curvature radius, optical properties of the materials, and, ultimately, by 2PP printing resolution.

## 4 Conclusions

A novel fabrication method for the realization of freestanding ultra-thin THz metasurfaces is described. The peculiarity of the technique is to merge the intrinsic ability of 2PP direct laser writing of creating dielectric structures with the flexibility of ultra-thin polymeric films to produce conformable plasmonic absorbers. Exploiting its spontaneous delamination in water, the polymeric nanomembrane can be in fact employed as freestanding support for the metallized 2PP printed metasurface structure. These devices were shown to present a relatively high level of absorption thanks to the built-in presence of two complementary metal planes arising in the structure after metallization. Upon creating a metal ground plane, achievable by coupling a second metal coated nanomembrane to the bottom of the structure, the devices can provide quasi-perfect resonant absorption. Importantly, owing to the ultra-thin thickness and high flexibility of the used polymeric material, the fabricated THz metasurface absorbers show unprecedented degree of conformability to high curvature surfaces. This is obtained through a water transfer process which allows all-round wrapping onto cylindrical surfaces with curvature as low as 12.5 μm in a straightforward and repeatable way. The proposed fabrication procedure can thus enable the realization of conformal metasurfaces for new lightweight, flexible or, eventually, wearable photonic devices. From a general perspective these devices can be suitable for integration in complex geometric systems, or they can be exploited for optical camouflage or information encryption in the emergent field of free-form optics. Strictly focusing on the THz frequency range, the extreme conformability combined with 3D geometrical flexibility of 2PP make them particularly beneficial for the realization of radar cross sectional reductors [48], and for the development of biosensors [49] in complex environments. Moreover, optical characterizations perfomed by TDS analysis show that the selected polymeric materials (PVF for the ultra-thin film and IP-Dip for the 2PP metasurface structure) have relatively low absorption losses and permittivity in the investigated range, revealing them as materials to be exploited at THz frequencies. Interestingly, the capacitor-like structure of the device created by the connected top metallization and bottom ground plane can be exploited in different ways: for example, to perform direct measurement of the capacitive dependence of the system upon illumination to form metasurface-based thermal detectors, or, it can also serve as gate to vary the EM response of the system to create dynamically tunable metasurfaces, provided that electric-field responsive material is integrated in the system.

In summary, owing to the presented novel fabrication method of freestanding highly conformable THz metasurfaces and for the new suitable materials introduced for this frequency range, this work can allow and ease the development of ultra-thin, flexible hybrid or all-dielectric THz devices which may boost the application of THz technology, and flexible photonics in general.

## Supplementary Material

Supplementary Material Details

## References

[1] Linden S., Enkrich C., Dolling G. (2006). Photonic metamaterials: magnetism at optical frequencies. IEEE J. Sel. Top. Quantum Electron..

[2] Veselago V., Braginsky L., Shklover V., Hafner C. (2006). Negative refractive index materials. J. Comput. Theor. Nanosci..

[3] Beh T. C., Kato M., Imura T., Oh S., Hori Y. (2012). Automated impedance matching system for robust wireless power transfer via magnetic resonance coupling. IEEE Trans. Ind. Electron..

[4] Landy N. I., Sajuyigbe S., Mock J. J., Smith D. R., Padilla W. J. (2008). Perfect metamaterial absorber. Phys. Rev. Lett..

[5] Mueller J. P. B., Rubin N. A., Devlin R. C., Groever B., Capasso F. (2012). Metasurface polarization optics: independent phase control of arbitrary orthogonal states of polarization. IEEE Trans. Ind. Electron..

[6] Walther B., Helgert C., Rockstuhl C. (2012). Spatial and spectral light shaping with metamaterials. Adv. Mater..

[7] Chen H. T., Taylor A. J., Yu N. (2016). A review of metasurfaces: physics and applications. Rep. Prog. Phys..

[8] Yu N., Capasso F. (2014). Flat optics with designer metasurfaces. Nat. Mater..

[9] Kamali S. M., Arbabi A., Arbabi E., Horie Y., Faraon A. (2016). Decoupling optical function and geometrical form using conformal flexible dielectric metasurfaces. Nat. Commun..

[10] Wang Y., Zhao C., Wang J. (2021). Wearable plasmonic-metasurface sensor for noninvasive and universal molecular fingerprint detection on biointerfaces. Sci. Adv..

[11] Liu X., Wang J., Tang L., Xie L., Ying Y. (2016). Flexible plasmonic metasurfaces with user-designed patterns for molecular sensing and cryptography. Adv. Funct. Mater..

[12] Burch J., Di Falco A. (2019). Holography using curved metasurfaces. Photonics.

[13] Wu K., Coquet P., Wang Qi J., Genevet P. (2018). Modelling of free-form conformal metasurfaces. Nat. Commun..

[14] Righini G. C., Krzak J., Lukowiak A., Macrelli G., Varas S., Ferrari M. (2021). From flexible electronics to flexible photonics: a brief overview. Opt. Mater..

[15] Walia S., Shah C. M., Gutruf P. (2015). Flexible metasurfaces and metamaterials: a review of materials and fabrication processes at micro-and nano-scales. Appl. Phys. Rev..

[16] Dhillon S. S., Vitiello M. S., Linfield E. H. (2017). The 2017 terahertz science and technology roadmap. J. Phys. D: Appl. Phys..

[17] Tao H., Bingham C. M., Strikwerda A. C. (2008). Highly flexible wide angle of incidence terahertz metamaterial absorber: design, fabrication, and characterization. Phys. Rev. B.

[18] Fan K., Zhao X., Zhang J. (2013). Optically tunable terahertz metamaterials on highly flexible substrates. IEEE Trans. Terahertz Sci. Technol..

[19] Cong L., Xu N., Gu J., Singh R., Han J., Zhang W. (2014). Highly flexible broadband terahertz metamaterial quarter-wave plates. Laser Photonics Rev..

[20] Li J., Shah C. M., Withayachumnankul W. (2013). Flexible terahertz metamaterials for dual-axis strain sensing. Opt. Lett..

[21] Zhang X., Wang Y., Cui Z. (2021). Carbon nanotube-based flexible metamaterials for THz sensing. Opt. Mater. Express.

[22] Ako R. T., Upadhyay A., Withayachumnankul W., Bhaskaran M., Sriram S. (2020). Dielectrics for terahertz metasurfaces: material selection and fabrication techniques. Adv. Opt. Mater..

[23] Khodasevych I. E., Shah C. M., Sriram S. (2012). Elastomeric silicone substrates for terahertz fishnet metamaterials. Nat. Protoc..

[24] Liu X., MacNaughton S., Shrekenhamer D. B. (2010). Metamaterials on parylene thin film substrates: design, fabrication, and characterization at terahertz frequency. Appl. Phys. Lett..

[25] Aksu S., Huang M., Artar A. (2011). Flexible plasmonics on unconventional and nonplanar substrates. Adv. Mater..

[26] Qin D., Xia Y., Whitesides G. M. (2010). Soft lithography for micro-and nanoscale patterning. Nat. Protoc..

[27] Meitl M. A., Zhu Z. T., Kumar V. (2006). Transfer printing by kinetic control of adhesion to an elastomeric stamp. Nat. Mater..

[28] Bückmann T., Stenger N., Kadic M. (2012). Tailored 3D mechanical metamaterials made by dip-in direct-laser-writing optical lithography. Adv. Mater..

[29] Seniutinas G., Weber A., Padeste C., Sakellari I., Farsari M., David C. (2018). Beyond resolution 100 nm in 3D laser lithography – post processing solutions. Microelectron. Eng..

[30] Harinarayana V., Shin Y. C. (2021). Two-photon lithography for three-dimensional fabrication in micro/nanoscale regime: a comprehensive review. Opt. Laser Technol..

[31] Den Hoed F., Ottomaniello A., Tricinci O., Cesarecciu L., Carlotti M., Raffa P., Mattoli V. (2023). Facile handling of two-photon polymerized 3D microstructures by ultra-conformable freestanding polymeric membranes. *Adv. Funct. Mater.*.

[32] Vendamme R., Onoue S. Y., Nakao A., Kunitake T. (2006). Robust free-standing nanomembranes of organic/inorganic interpenetrating networks. Nat. Mater..

[33] Jiang C., Markutsya S., Pikus Y., Tsukruk V. V. (2004). Freely suspended nanocomposite membranes as highly sensitive sensors. Nat. Mater..

[34] Fujie T., Ricotti L., Desii A., Menciassi A., Dario P., Mattoli V. (2011). Evaluation of substrata effect on cell adhesion properties using freestanding polymeric nanosheets. Langmuir.

[35] Fujie A. D., Ventrelli L., Mazzolai B., Mattoli V. (2012). Inkjet printing of protein microarrays on freestanding polymeric nanofilms for spatio-selective cell culture environment. Biomed. Microdevices.

[36] Redolfi Riva E., Desii A., Sartini S., La Motta C., Mazzolai B., Mattoli V. (2013). PMMA/polysaccharides nanofilm loaded with adenosine deaminase inhibitor as a platform for targeted anti-inflammatory drug delivery. Langmuir.

[37] Ridolfi Riva E., Desii A., Sinibaldi E. (2014). Gold nanoshell/polysaccharides nanofilm for controlled laser-assisted tissue thermal ablation. ACS Nano.

[38] Taccola S., Greco F., Zucca A. (2013). Characterization of free-standing PEDOT:PSS/iron oxide nanoparticles composite thin films and application as conformable humidity sensors. ACS Appl. Mater. Interfaces.

[39] Taccola S., Greco F., Mazzolai B., Mattoli V., Jager E. W. H. (2013). Thin film free standing PEDOT:PSS/SU8 bilayer microactuators. J. Micromech. Microeng..

[40] Viola F. A., Barsotti J., Melloni F. (2021). A sub 150 nanometers thick and ultra-conformable solution processed all-organic transistor. Nat. Commun..

[41] Greco F., Zucca A., Taccola S., Mazzolai B., Mattoli V. (2013). Patterned free-standing conductive nanofilms for ultra-conformable circuits and smart interfaces. ACS Appl. Mater. Interfaces.

[42] Barsotti J., Hirata I., Pignatelli F., Caironi M., Greco F., Mattoli V. (2018). Ultraconformable freestanding capacitors based on ultrathin polyvinyl formal films. Adv. Electron. Mater..

[43] Baxamusa S. H., Stadermann M., Aracne-Ruddle C. (2014). Enhanced delamination of ultrathin free-standing polymer films via self-limiting surface modification. Langmuir.

[44] Watts C. M., Liu X., Padilla W. J. (2012). Metamaterial electromagnetic wave absorbers. Adv. Mater..

[45] Withayachumnankul W., Abbott D. (2009). Metamaterials in the terahertz regime. IEEE Photonics J..

[46] Tao Hu, Landy N. I., Bingham C. M., Zhang X., Averitt R. D., Padilla W. J. (2008). A metamaterial absorber for the terahertz regime: design, fabrication and characterization. Opt. Express.

[47] Wienold M., Schrottke L., Giehler M., Hey R., Anders W., Grahn H. T. (2009). Low-voltage terahertz quantum-cascade lasers based on LO-phonon-assisted interminiband transitions. Electron. Lett..

[48] Yin W., Shen Z., Li S. (2022). Flexible broadband terahertz absorbers for RCS reduction on conformal surfaces. Opt. Commun..

[49] Yin W., Shen Z., Cui Y. (2022). Highly sensitive terahertz sensing with 3D-printed metasurfaces empowered by a toroidal dipole. Opt. Lett..

