# Supplementary material for: Highly conformable terahertz metasurface absorbers via two-photon polymerization on polymeric ultra-thin films
Source: Nanophotonics. 2023 Feb 20;12(8):1557–70. doi: 10.1515/nanoph-2022-0667 (PMC11501486; doi:10.1515/nanoph-2022-0667)
Supplement: Supplementary file 1 — Supplementary Material Details [file j_nanoph-2022-0667_suppl.pdf]

Research Article

Andrea Ottomaniello*, Paolo Vezio, Omar Tricinci, Frank M. Den Hoed, Paul Dean, Alessandro Tredicucci, and Virgilio Mattoli*

Highly conformable terahertz metasurface absorbers via two-photon polymerization on polymeric ultra-thin films

Supplementary materials

https://doi.org/10.1515/sample-YYYY-XXXX
Received Month DD, YYYY; revised Month DD, YYYY; accepted Month DD, YYYY

1 Fabrication procedure details

As described in the main text of the manuscript, the fabrication procedure can be divided in six fundamental steps:

– **Polivynil formal membrane preparation**: the polivynil formal (PVF) film preparation relies on a spin-coating procedure developed by Baxamusa et al. [1] and subsequently exploited by IIT to fabricate conformable freestanding capacitors based on thin PVF films [2]. PVF from Sigma-Aldrich (trade name Vinylec K) Ethyl lactate (EL, $\geq 98\%$, FCC, FG) and PDAC (20%wt in water solution) are used to perform spin-coating on polished surface of a 2 inches diameter (381 ± 25 μm-thick) silicon wafers by Silicon Materials (Si-Mat). A PDAC solution of 0.5 %wt is prepared by diluting the commercial 20%wt solution with deionized water. The PVF solution of 1.5% in weight is prepared by dissolving PVF in EL. Solutions are stirred at 650 rpm at a temperature fo 50°C until all PVF is dissolved. PVF solution are filtered immediately before use with a hydrophobic filter (0.2 μm pores diameter, Minisart). For each prepared device, two silicon wafers are first functionalized with a subnanometric PDAC layer. After first performing a plasma oxygen treatment to enhance the silicon wafers wettability (60 W, 1 min), about 2 mL PDAC solution is deposited and spin-coated on each of them at 4000 rpm for 15 s. The wafers are then baked at 100°C for 10 s on a hotplate. By rinsing them with deionized

***Corresponding author: Andrea Ottomaniello,** Center for Materials Interfaces, Istituto Italiano di Tecnologia, Via R. Piaggio, 34, 56025 Pontedera, PI, Italy, e-mail: andrea.ottomaniello@iit.it
Paolo Vezio, Dipartimento di Fisica E. Fermi, Università di Pisa, Largo Pontecorvo 3, 56127 Pisa (Italy), e-mail: paolo.vezio@df.unipi.it
Omar Tricinci, Center for Materials Interfaces, Istituto Italiano di Tecnologia, Via R. Piaggio, 34, 56025 Pontedera, PI, Italy, e-mail: omar.tricinci@iit.it
Frank M. Den Hoed, Engineering and Technology institute Groningen (ENTEG), University of Groningen, Nijenborgh 4, Groningen, 4747 AG, The Netherlands, and Center for Materials Interfaces, Istituto Italiano di Tecnologia, Via R. Piaggio, 34, 56025 Pontedera, PI, Italy, e-mail: frank.denhoed@iit.it
Paul Dean, School of Electronic and Electrical Engineering, University of Leeds, Leeds LS29JT, UK, e-mail: P.Dean@leeds.ac.uk
Alessandro Tredicucci, Dipartimento di Fisica E. Fermi and Center for Instrument Sharing of the University of Pisa (CISUP), Università di Pisa, Largo Pontecorvo 3, 56127 Pisa (Italy), e-mail: alessandro.tredicucci@unipi.it
***Corresponding author: Virgilio Mattoli,** Center for Materials Interfaces, Istituto Italiano di Tecnologia, Via R. Piaggio, 34, 56025 Pontedera, PI, Italy, e-mail: virgilio.mattoli@iit.it

water for a few seconds in order to dissolve the PDAC residues, the wafers are dried with a compressed air gun. After this process the wafer surface looks homogenous with almost unchanged color as only a sub-nanometer functionalization is performed. The PVF solution is then spin-coated over PDAC sub-nm layer in a two-step process of a total duration of 10 s. During the first 5 s, spin-coating speed is set at 300 rpm while during the remaining 5 s it is set at 3000 rpm. About 2 mL PVF solution is deposited for each nanosheet spinning session, and it is then baked for 60 s at 50°C on a hotplate. The PVF nanosheets thickness measurement is performed by means of a P-6 stylus profilometer (KLA Tencor, USA) averaging over 10 measured values randomly acquired on the film surface before the release from silicon-wafer. For the PVF concentration used the measured thickness is equal to 50 ± 2 nm.

- **2PP printing of the metasurface**: Direct laser printing (DLW) of the dielectric structure of the device is performed by 2PP using the Photonic Professional GT-2 (from Nanoscribe) using the commercial 2PP photoresin IP-Dip (Nanoscribe) in dill configuration with a 63x objective. The PVF spin-coated silicon wafer provides a clear interface for the 2PP printing whose bottom plane is fixed at 0.5 μm underneath the silicon interface. Three-axis plane focus alignment is performed in order to check and eventually control the planarization of the substrate surface to achieve a homogenous thickness of the printed structure across its size. The laser power and beam scanning velocity of the beam are fixed to 40 W and 10.000 μm/s for all the realized printing. The slicing and hatching distant are fixed at 0.3 and 0.3 μm, respectively. The metasurface geometry is build using the Nanoscribe slicer software by periodically repeating as square array the unit cell cad with pitch exactly equal to its size in order to have a connected structure. A nominal thickness of the structure is calibrated to produce an offset on the experimental measured one of $+1.0 \pm 0.2$ μm. The development is performed completely immersing the silicon wafer in PGMEA for 10 min, then the wafer is rinsed by spraying several times IPA on the wafer surface until no photoresin residues are visible, and, finally, it is dried with a dry air gun.

- **Top gold metallization**: The 50 nm-thick layer of gold providing both the top and bottom metallization of the structure is obtained by magnetron sputtering deposition. The deposition process is performed with a power of 10 W, at an argon pressure of $\sim 2.1 \times 10^{-2}$ mbar and with constant deposition rate of 2.5 Å/s. For a 50 nm-thick PVF film, a 50 nm-thickness of the gold layer is found as the maximum depositable thickness which does not affect the repeatability of the PVF delamination process. Higher deposited gold thicknesses can be achieved with higher thickness of the PVF film. Thermal evaporation of the same thicknesses instead results in the compromise of the delamination process due to the temperature reached by the substrate in the process. On both PVF spin-coated silicon wafers, a 3×3 mm^2 gold pad is realized using a 0.5 mm-thick plastic shadow mask where the aperture was made using a versa-laser. In the case of the wafer with the realized 2PP structure, this aperture is simply aligned to the latter by hands.

- **Delamination process**: The region of the PVF film to be delaminated from the silicon wafer are outlined using a doctor blade. A 1.5×1.5 cm^2 cut centering the device area is performed in the PVF layer. This size is made to collect the PVF film on a holed plexiglass frame with internal and external diameter of 1 and 1.8 cm and thickness 3 mm. Operatively, the silicon wafer is progressively inserted in bath water with a $\sim 45°$ angle at a speed which allows the film to spontaneous delaminate without forming excessive stress on the film (fraction of mm/s). Figure S1a and b show a representative device during delamination.

- **Collection process**: As the film is completely released and floating on the water surface it can be collected from the top via the holder by handling it with standard tweezers. Once centered the frame on the metallized region of the film, the latter is able to attach via Van der Waals forces to the frame borders. During the pick up rotational movement the water tension allows to perfectly flat the film and ensure its adhesion on the frame. The film is then left under a hood to dry. The collection process for a representative device is shown in Figure S1c and d.

– **Bottom metallization**: The collection process is first performed for the film carrying the metallized metasurface. When ready, the same holder is used to collect the second delaminated PVF layer carrying the gold pad which finally provides the bottom metallization with the same procedure.

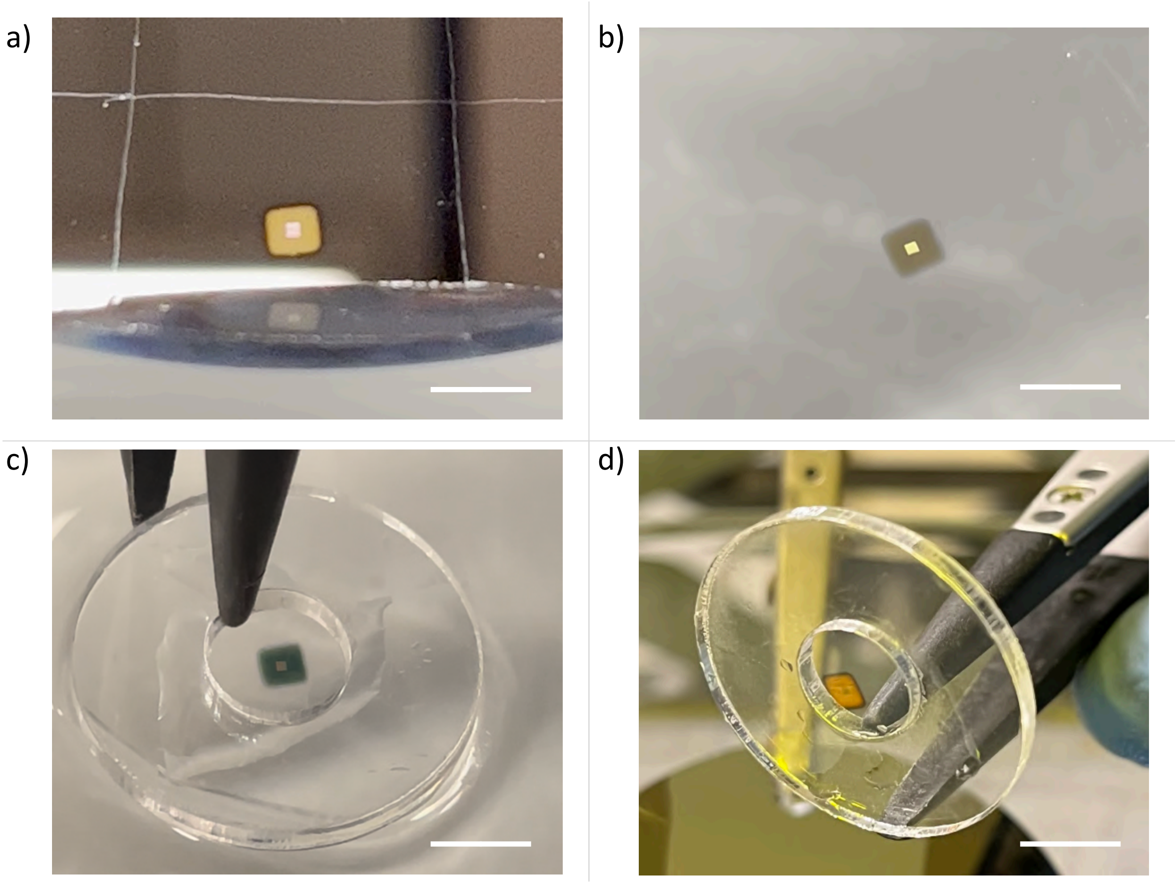

Fig. 1: Images of a sample during the delamination and collection steps of the fabrication. a) A silicon wafer is immersed in water to delaminate the PVF spincoated layer on which the 2PP printing and metallization of a metasurface was previously performed. b) the delaminated PVF floating on water and carrying the metallized metasurface. c) Device during the collection on a holed frame. d) Free-standing device after collection. The scale bar is 6 mm for each image.

2 Details of the simulations

The calculations shown both in the manuscript and in the Supplementary materials are obtained through finite-element simulations using the commercial software COMSOL Multiphysics. The simulated full-wave propagation of electro-magnetic (EM) waves through the system is obtained using the electromagnetic (EM) waves modulus.

The three-dimensional domain is built as a parallelepiped unit-cell constituted along its out-of-plane direction by: the central metasurface structure via an extruded work-plane geometry to provide the dielectric thickness, four 50 nm-thick layers (one above and three below the metasurface) representing the metallization and polymer film, the top and bottom 100 μm medium domains and two perfectly matched layers (PML), above and below the entire domain to provide an infinite extension of the external medium in the vertical direction. Three-different ports are inserted: one exciting port 90 μm above the metasurface structure, and

two analyzing ports at the interfaces between the PML boxes and the external medium domains, one above and one below the metasurface to detect the reflected and transmitted EM wave, respectively. In all ports the same linear polarization is fixed. Floquet-boundary conditions are applied to all later boundaries in order to provide the in-plane unit-cell periodicity of the metasurface structure.
The top in-plane boundary of the metasurface is meshed as free triangular with the following element parameters: maximum and minimum size equal to 1 and 0.05 μm, respectively, with a maximum growth rate of 1.1. The metasurface domain is meshed by swepting the top boundary mesh to the bottom boundary of the metasurface structure (i.e. the dielectric layer) with a 10-points distribution. The lateral boundaries of the external medium are meshed as free-triangular with maximum and minimum element size of 5 and 0.5 μm, respectively. Excluding the PML boxes, all remaining domains are meshed as free-tetrahedral with maximum and minimum element size of 4 and 0.5 μm, respectively. This mesh structure is used for both THz simulations and those performed in the 20-32 THz frequency shown in the following section. In the latter, the size of the elements are just scaled according to the scaling of the unit-cell. Exploiting the described mesh, a minimum and average element quality of 0.61 and 0.94 are obtained, respectively, which ensure a very good spatial resolution for the EM distribution calculated at a specific frequency. The frequency step of the simulations is fixed to 0.02 THz.

3 Additional characterization of THz metasurface aborbers

In order to characterize the fabricated metasurface absorbers in reflection configuration, we used a THz-FTIR (Jasco) equipped by a Hg-lamp as source and a He-cooled bolometer as detector. Even if this set-up was not able to provide a reflection spectrum at normal incidence, we were able to measure the reflectance of the samples at around 45° angle of incidence in the frequency range from 2 to 5 THz by exploiting a system of flat and parabolic mirrors to focus the unpolarized THz light onto the metasurface. The measured spectrum for a double-metal metasurface with 43 μm unit-cell is reported in Figure S2 with a resolution of 4 cm^{-1}. The simulated resonance at 3.5 THz for the same metasurface at normal incidence reported in the main manuscript in Figure 4 is observed to slightly blueshift to 3.7 THz, while other two resonances emerge at the borders of the investigated frequency range. This is in agreement with the simulations reported in the same figure obtained with linearly polarized light at oblique incidence. As we excited the metasurface with unpolarized light, the metasurface electromagnetic response at oblique incidence is found to present an average behaviour between the calculated spectrum for transverse magnetic and transverse electric polarizations.

4 Characterization of devices in the 20-34 THz range

The optical properties of the used polymeric materials were also studied at higher frequencies with respect to the THz range. In Figure S3a and b the transmittance, reflectance and absorbance of a 400 nm-thick PVF film and a 3.6 μm-thick IP-Dip layer are shown, respectively. They were measured using a micro-FTIR (Jasco) equipped by an optical microscope which allowed to perform spectroscopy with a few μm-resolution. In the reported measurements an area of 200×200 μm^2 was fixed. In the investigated frequency range (20-34 THz), the PVF film shows a transmittance $T \geq 95\%$ and an absorbance $A \leq 2\%$, apart from a resonance feature at $\sim$ 30.5 THz where T and A decreases/increases to 90% and 4%, respectively. The IP-Dip layer spectra instead present peculiar absorption resonances at approximately 22.5, 24.3, 29.6 and 31.9 THz with absorption levels of about 13.1, 20, 37.6 and 53.4%, respectively. As shown by the reflection spectrum in a broader frequency range (20-200 THz) (see inset of Figure S3b), these absorption amplitudes are affected (enhanced) by the presence of Fabry-Perot oscillations arising from the particular characterized sample. Nevertheless, their periodicity allows to calculate a refractive index of $\sim$ 1.6.

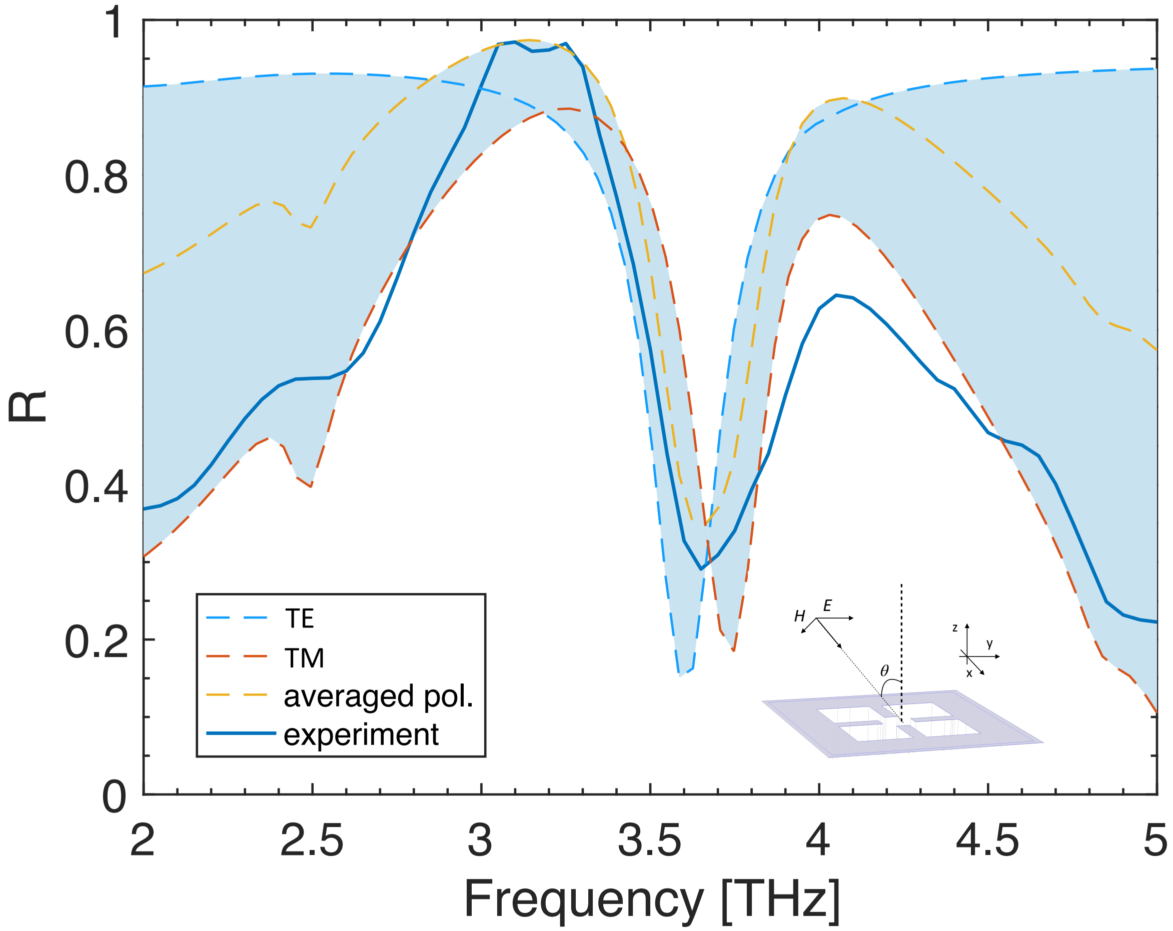

Fig. 2: a) Reflectance spectrum at $45°$ angle of incidence (blue curve) obtained using a THz-FTIR equipped with a Hg-lamp and a He-cooled bolometer. Finite-element simulations at the same incidence angle for transverse electric (TE) and transverse magnetic (TM) polarizations, represented by a blue and a red dashed curve, respectively. The yellow dashed curve is the average between the reflectances of the two polarizations, while the light blue colored area highlights the reflectance values in between the two calculated metasurface responses.

Free-standing metasurface devices were fabricated to present LC-resonance absorption in this investigated frequency region. The absorbances of three metasurface absorbers with 7, 6 and 5 μm with only the single top metallization are shown in Figure S3c. They are obtained by a simple geometric scaling of the unit-cell of the metasurfaces reported in the main text. The thickness for all metasurfaces is 1.5 μm. They show absorption peaks which blueshift accordingly to the scaling of the metasurface unit-cell, and which all present absorption amplitudes $\geq 50\%$. With respect to the single-metal THz metasurfaces shown in the main manuscript, those reported here show higher absorption levels due to to the higher absorption of IP-Dip in this frequency range. Nevertheless, a clear enhancement in the absorption caused by the presence of the metasurface resonator is measured as well. Finite-element simulations (dashed lines in 3c) reveal also in this case a good agreement with the experimental data. Both amplitudes and frequency of the absorption peaks are reproduced. The Q-factor discrepancy between simulation and experimental results are again attributed to the possible deposition of a thin layer of gold on the metasurface side-walls due to the sputtering deposition process. In these calculations, the optical properties for the two polymeric materials are retrieved/approximated from those presented in Figure S3a-b or extended by linear interpolation from those reported in the main manuscript. The real part (n) and imaginary (k) are set to $n = 2.5$ and $k = 0.003$ for the PVF layer, while those for the IP-Dip layer are fixed to $n = 1.6$ and $k = 0.03$. The gold refractive index is again fixed to $n = 220$ and $k = 310$.

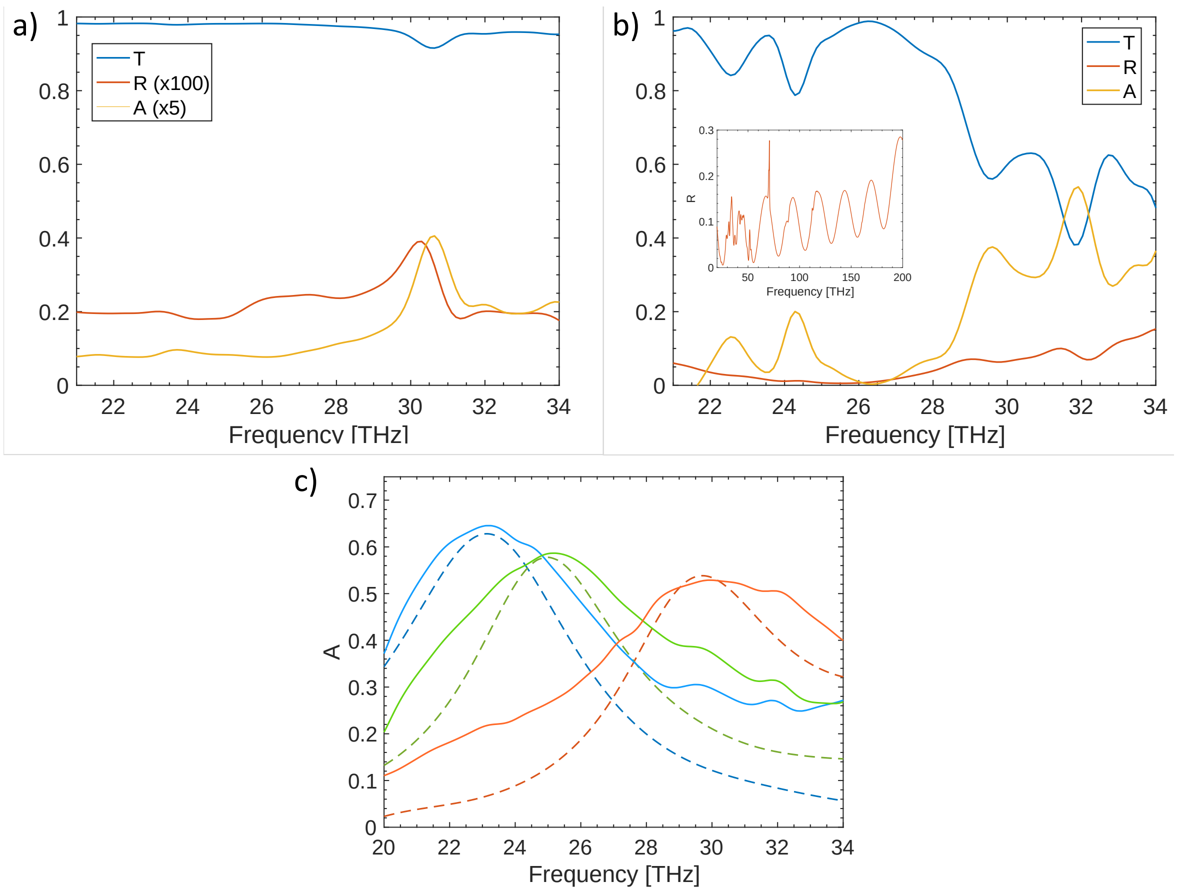

Fig. 3: Optical characterization of PVF, IP-Dip and metasurface absorbers. Transmittance, reflectance and absorbance in the $21-34$ THz-range of a 400 nm-thick PVF film and a 3.6 μm-thick IP-Dip layer are shown in (a) and (b), respectively. The inset of (b) shows the reflection of IP-DIP in the range $20-200$ THz range to highlight the Fabry-Perot resonance of the sample. (c) Absorption for three metasurfaces having only the top metallization with 7, 6 and 5 μm unit-cell represented by the orange, green and red solid curves, respectively. Dashed lines with same darker colors report the corresponding calculated results obtained via full-wave finite-element simulations.

Optical images of a representative fabricated metasurface suspended via a 50 nm-thick polymeric membrane on a circular holed frame are reported in Figure S4.

The conformability performance of the fabricated devices were then investigated by transfer the metasurfaces on cylindrical objects with different radius of curvature. Examples of conformal transfer (achieved with the water transfer technique described in the main manuscript) on a fabric wire of 200 μm-radius, and onto metal wires up to a minimum radius of 12.5 μm are shown in Figure S5. We limited the investigation up to this radius to have at least a few unit-cells of the metasurface along a semi-circunference of the wire, still guaranteeing the overall response of the metasurface in the explored frequency range. This demonstrates that the degree of conformability of the metasurfaces developed by this technique is limited only by the geometric constraints (unit-cell size and thickness), optical losses of the materials (depending on the frequency range), and, ultimately, by the 2PP printing resolution. The EM response of the single-metal 6μm unit-cell metasurface depending on the radius of curvature was measured in reflection configuration via μ-FTIR apparatus. For each device, the reflection spectrum was acquired by averaging 10 measurements focusing on a square surface with size equal to the corresponding diameter of the conformed wire. The measured reflection spectra are shown in Figure S5e. The radius of curvature of the metasurface can be observed to not clearly affect the peak frequency and amplitude of the resonance, while a progressive increase (decrease) of the reflectance it is observed at frequencies higher (lower) than the center resonance frequency.

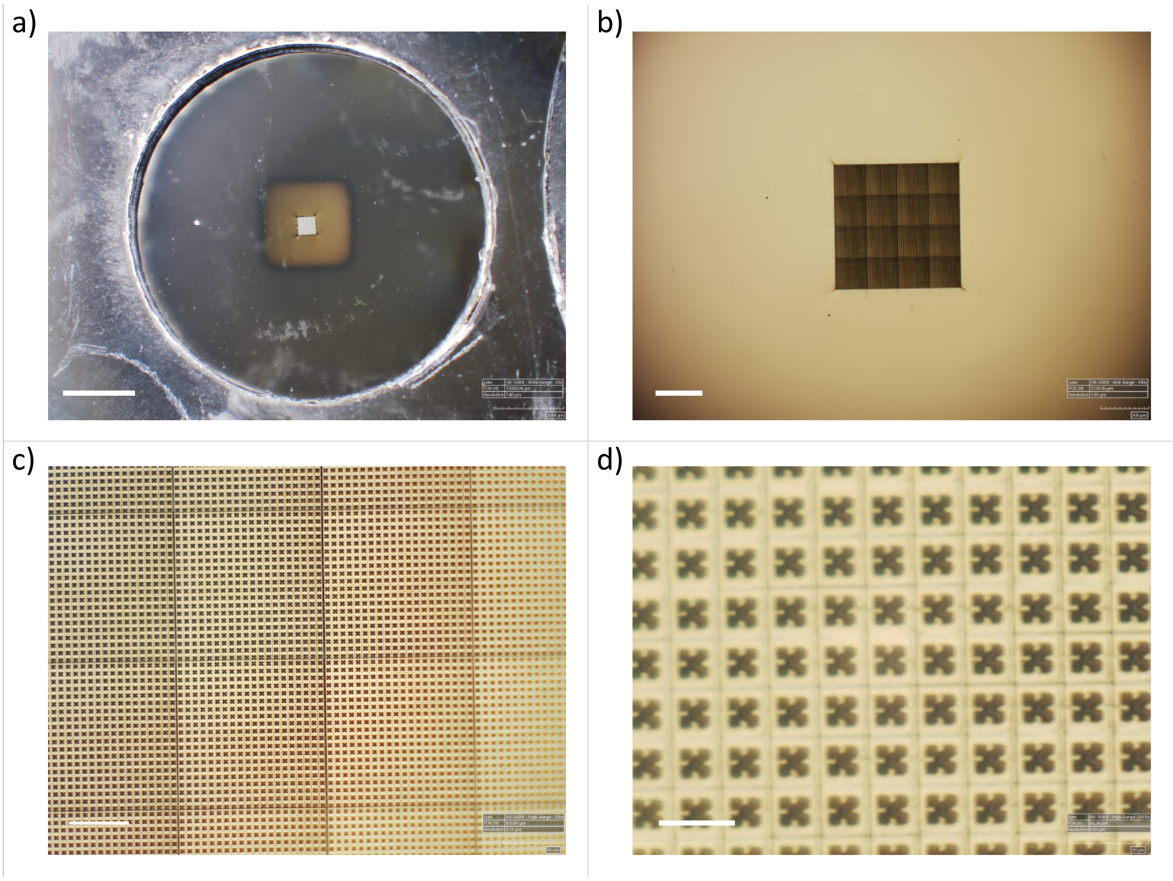

Fig. 4: Optical images of a conformable metasurface absorber with a 6 μm unit- cell and single top metallization. a) Free-standing absorber on the 50 nm-thick PVF layer after delamination and collection on a holed frame. b) Image of the 600×600 μm^2 central region of the sample where the metasurface is located. (c) and (d) Zoom x700 and x5000 of the metasurface, respectively. Scale bars in (a), (b), (c) and (d) are 2000, 200, 50 and 10 μm, respectively.

References

[1] Baxamusa, Salmaan H and Stadermann, Michael and Aracne-Ruddle, Chantel and Nelson, Art J and Chea, Maverick and Li, Shuali and Youngblood, Kelly and Suratwala, Tayyab I, "Enhanced delamination of ultrathin free-standing polymer films via self-limiting surface modification," *Langmuir*, vol. 30, no. 18, 2014, pp. 5126–5132.https://doi.org/10.1021/la5011665

[2] Barsotti, Jonathan and Hirata, Ikue and Pignatelli, Francesca and Caironi, Mario and Greco, Francesco and Mattoli, Virgilio, "Ultraconformable freestanding capacitors based on ultrathin polyvinyl formal films," *Advanced Electronic Materials*, vol. 4, no. 11, 2018, pp. 1800215. https://doi.org/10.1002/aelm.201800215

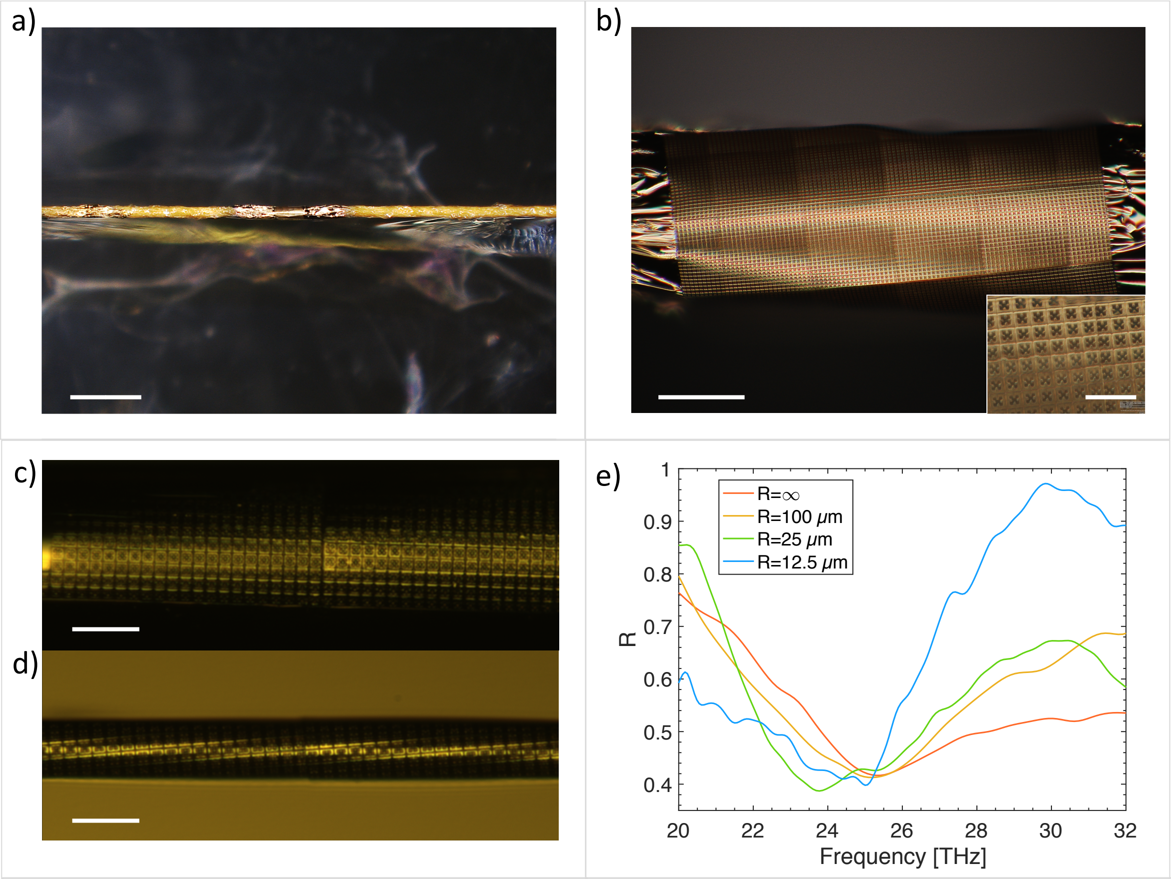

Fig. 5: (a) Optical image of a 200 μm-radius fabric wire with a 6 μm-unit cell metasurface absorber wrapped around it. a) 700x zoom of the transferred metasurface. Inset: 5000x zoom. Scale bars in (a), (b) and its inset are 2000, 300, and 20 μm, respectively. c) and d) show the same metasurface wrapped on a wire of 50 and 25 μm radius, respectively. e) Comparison between the reflection spectum of a free-standing 6 μm-unit cell metasurface ($R = \infty$)with those of three metasurfaces of the same size transferred on wires with different radius of curvature, $R = 50$, 25 and 12.5 μm.
